# Modified Carnoy’s Versus Carnoy’s Solution in the Management of Odontogenic Keratocysts—A Single Center Experience

**DOI:** 10.3390/jcm12031133

**Published:** 2023-02-01

**Authors:** Anna Janas-Naze, Wei Zhang, Mariusz Szuta

**Affiliations:** 1 Department of Oral Surgery, Medical University of Lodz, 92-213 Lodz, Poland; 2 Shanxi Oral Disease Prevention and Treatment Center, Shanxi Provincial People’s Hospital, Taiyuan 030012, China; 3 Department of Oral Surgery, Jagiellonian University Medical College, 31-008 Cracow, Poland

**Keywords:** Carnoy’s solution, modified Carnoy’s solution, odontogenic cysts, odontogenic keratocyst, recurrence

## Abstract

To date, few studies have been conducted to test the effectiveness of Carnoy’s (CS) versus modified Carnoy’s (MC) solution for preventing the recurrence of odontogenic keratocysts, which are potentially aggressive lesions. To evaluate the efficacy of MC application, we conducted a retrospective cohort study over an 18-year period, from October 2004 to October 2022, in 122 patients treated surgically with adjunctive chemical cautery, with either CS (n = 73; median age: 30 years) or MC (n = 49; median age: 42 years), by a single surgeon. The primary outcome variables were observed recurrence and interval to recurrence. Independent variables were demographics, location, clinical presentation at baseline, adjacent tooth extraction, and bone grafting. Males predominated in both groups. No statistically significant differences were observed between the two arms in terms of recurrences in particular months, with six patients (8.2%) in the CS arm and 5 (10.2%) in the MC arm. Of the 11 recurrences, 10 were observed within the first 2 years post-surgery, with only one occurring in the 7th year of follow-up. Thus, when used as adjunctive therapy, the application of MC has an efficiency comparable to that of CS for lowering the recurrence rate of odontogenic keratocysts.

## 1. Introduction

Odontogenic keratocysts (OKC), although classified as benign uni- or multicystic lesions, are known for their aggressive and infiltrative properties. In particular, with the change in terminology from keratocystic odontogenic tumor (KCOT), some clinicians may have lost vigilance when faced with such a probable diagnosis. Several studies [1,2,3] have reported aggressiveness, in which the OKCs were found to penetrate the temporalis or masseter muscle and even the mediastinum. The ability of OKCs to extend into the surrounding tissues significantly impedes entire enucleation, thus leading to imminent recurrence. Stoelinga [4,5] demonstrated the existence of nests composed of epithelial cells with coexisting microcysts observed in the mucosa surrounding the OKCs. Over the last few decades, numerous studies have emphasized the risk of recurrence following surgical treatment, regardless of the type of surgery. Interestingly, several studies [6,7,8,9] have described the possible recurrence of OKCs in a bone graft, which could be viewed as further confirmation of the overlying mucosa being the origin of the newly formed cysts.

According to several studies [10,11,12], the recurrence rate ranges from 13% to as high as 58% in cases of sole surgical enucleation, curettage, and peripheral ostectomy. Attempts have been made to reduce this astonishingly high rate through the introduction of adjuvant therapy in the form of chemical cauterization using Carnoy’s solution (CS). CS is primarily known as a fixative agent in histopathology; however, its cauterization features first resulted in its utilization for the treatment of cysts 90 years ago by Cutler and Zollinger [13]. CS comprises 3 mL of chloroform, 6 mL of ethanol, 1 mL of glacial acetic acid, and 1 g of ferric chloride [14]—a combination that induces chemical necrosis to a depth of 1.5 mm, eliminating epithelial remnants and microcysts around the cyst wall. However, CS should not be applied to the bone cavity for a period exceeding 5 min, due to possible nerve or vessel injuries, with the most beneficial period being 3 min. If used with caution, CS is fairly safe, with the most common complications being infection, development of bony sequestra, and neuropathies, which are mostly caused by exceeding the critical time of exposure. 

The FDA banned the use of CS in 2013 as it contained chloroform, the use of which was banned in the United States due to its carcinogenic properties, as evidenced in animal studies [15]. Consequently, CS was replaced by the non-chloroform-containing modified Carnoy’s solution (MC). Even so, as shown by Ecker et al. [16], who performed a web-based survey among the members of the American Association of Oral and Maxillofacial Surgeons in 2015, CS continued to have significance in the treatment of OKCs, with 56% of surgeons still applying it despite the ban. To date, limited studies have been conducted to test the effectiveness of CS versus MC in preventing OKC recurrence, with inconclusive results: a search of PubMed and Medline databases using the keywords “Carnoy’s solution” OR “Modified Carnoy’s solution” AND “Odontogenic keratocyst” OR “Keratocystic odontogenic tumor” yielded only two relevant studies [17,18], with contradictory results. 

Therefore, to resolve the debate regarding the effectiveness of CS vs. MC, we decided to conduct a retrospective (due to the chloroform ban) study, with the null hypothesis that there would be no difference in the efficacy of CS and MC in preventing OKC recurrence. 

## 2. Materials and Methods

### 2.1. Study Design

To comprehensively conduct the planned research, a retrospective cohort study was carefully planned to avoid selection bias, which can substantially influence the results of studies with a retrospective nature. The study was conducted in the Department of Oral Surgery, Medical University of Lodz, Poland, in compliance with the Helsinki Declaration, following the acquisition of informed consent from the participants or their legal guardians and after obtaining approval from the Bioethical Committee of the Medical University of Lodz. The study was conducted over a period of 18 years, from October 2004 to October 2022. 

### 2.2. Patient Selection

The study population comprised patients treated surgically with adjunctive chemical cautery using either CS or MC by a single surgeon.

Inclusion criteria:

- Presence of histopathologically diagnosed parakeratinized OKC in maxilla or mandible
- Unilocular lesions
- Nonsyndromic OKC
- Capacity for giving consent
- Signed informed consent from the patient or guardian
- Patients who underwent surgeries (enucleation, curettage, peripheral osteotomy and subsequent CS or MC application) and completed the follow-up by October 2022
- Follow-up period of at least 5 years.

Exclusion criteria:

- Cases with incomplete clinical, radiological, or histopathologic data
- Patients with history of previous surgeries and recurrence of OKC
- Cases in which marsupialization was mandatory due to large size, cortical perforation, or proximity of the sinus membrane
- Cases in which segmental resection was performed due to severe bone resorption or pathological fracture
- Patients diagnosed with Gorlin’s syndrome or nevoid basal cell carcinoma syndrome.

### 2.3. Intervention

All surgeries were performed by a single surgeon (AJN) specializing in oral surgery using the same surgical technique for all patients, which included enucleation and curettage of the lesion with elective extraction of the indicated teeth, followed by peripheral ostectomy using a round bur on a high-speed hand piece and 3 min application of either CS or MC using Kittner sponges or surgical cottonoids, depending on the cavity size and location. The clotted blood and necrotic tissue were subsequently scraped and washed with saline, followed by excision of the overlying mucosa. If the patient was scheduled for bone grafting, the procedure was performed subsequently.

### 2.4. Statistical Analysis

SPSS Statistics 25 software (IBM SPSS Inc., Armonk, NY, USA) was used for statistical analyses. The chi-squared test was used to assess statistical significance of correlation among the nominal variables, whereas the effect size was measured using the V Cramer of Phi coefficient. The Mann–Whitney U test was used to analyze the statistical significance of the differences between two independent groups. A *p*-value of less than 0.05 was considered statistically significant for all analyses.

## 3. Results

### Patient Characteristics

In total, there were 167 possibly eligible cases. Unfortunately, 18 of these patients were excluded because they were lost to follow-up: 23 were excluded due to an insufficient follow-up period, and four were ruled out due to a diagnosis of Gorlin syndrome. The data obtained from 122 patients who met the inclusion criteria were finally included in the analysis.

Of the 122 included patients, 73 (59.8%) were in the CS arm and 49 (40.2%) were in the MC arm. The two groups showed some differences (χ^2^(1) = 4.72; *p* = 0.03) but did not differ in terms of age (U = 1734.5; *p* = 0.78). The median age was 30 years in the CS group and 24 years in the MS group. The total cohort consisted of 42 women (34.5%) and 80 men (65.5%), with a 2:1 male-to-female ratio and a strong male predilection in both groups. 

Among the parameters listed in Table 1, bone grafting was statistically significantly more frequently performed in the CS group. However, the effect size indicated that this variable accounted for only a small difference between the groups. Further differences concerned the applied treatments.

The second analysis (Table 2) concerns recurrences in both arms. No statistically significant differences were observed between the arms in terms of recurrence in particular months. Interestingly, among the 11 recurrences, 10 were observed within the first 2 years post-surgery, with only one occurring later, in the 7th year of follow-up.

There were no recurrences observed in the CS arm in the period of 120 to 216 months post-surgery.

We found no statistically significant correlation between the variables analyzed against recurrences in various months. Nevertheless, the hypothesis that the number of recurrences increased with time was tested using the V Crammer coefficient. This analysis revealed a statistically significant correlation between the number of recurrences and the time point in the CS arm (V_cr_ = 0.15; *p* = 0.02), whereas no such correlation was found in the MC arm (V_cr_ = 0.15; *p* = 0.23). 

Subsequently, the number of recurrences during the whole follow-up period was summed, but no statistically significant correlation was observed (χ^2^(1) = 0.14; *p* = 0.75); there were six (8.2%) patients with recurrence in the CS arm and five patients (10.2%) in the MC arm.

Because only single recurrences were observed at 12-, 24-, 36-, 48-, and 84-months post-surgery, the statistically significant association between recurrences and all other variables was not tested.

However, a statistically significant correlation between the total number of recurrences in both arms and other explanatory variables was tested, as the number of overall recurrences in each group was at least five, which is the minimal sample size needed for adequate analysis. In the CS arm (Table 3), no statistically significant correlation was observed; in other words, the total number of recorded recurrences was similar for individual variables.

In the MC arm (Table 4), a statistically significant association was observed in the case of lesion location, with a higher percentage of recurrences observed in the maxilla. However, the results should be considered with caution because of the small number of observed recurrences in both groups.

Other analyses did not show statistically significant differences in terms of the age of patients who suffered from recurrences in either group (CS arm: U = 162; *p* = 0.43; MC arm: U = 89; *p* = 0.49).

The median age of the patients with observed recurrences was 17 years in the CS group and 24 years in the MC group. In cases where recurrences were not recorded, the ages were 32 years and 23.5 years, respectively.

No other statistically significant differences were observed, likely because of the low number of recorded recurrences. For this reason, no further advanced methods of statistical analysis, such as logistic regression, were used.

## 4. Discussion

In our evaluation of the performance of CS and MC for preventing the recurrence of OKCs, we found no statistically significant correlation between CS or MC and recurrences during the follow-up period. 

Our finding was consistent with the results presented by Donnelly et al. [17], who retrospectively analyzed 77 patients (36 in the CS arm and 41 in the MC arm), treated using the same surgical modality by a single surgeon. However, contrary to their findings, we did not observe a statistically significant association between preserving one or more teeth adjacent to the lesion and a higher percentage of recurrences. As mentioned before, this conclusion must be approached with caution because of the relatively low percentage of overall recurrences. 

The findings of our study contrast with those of Dashow et al. [18], who analyzed a retrospective cohort of 80 patients and reported a significantly lower recurrence rate in the CS arm as compared to the MC arm (11% vs. 36%). The reason for such discrepant findings is not clear, but it may involve the lack of surgical standardization, as Dashow et al.’s study involved three operating surgeons. The level of experience and different techniques may have been key factors in obtaining different results. Another factor may have been the histological features of the cysts, as the authors did not mention which variants of OKCs were included in their study. 

When discussing high recurrence rates regarding the evaluation of treatment modalities, we should consider determining the source of recurrence. A systematic review performed by Kaczmarzyk et al. [12] on the recurrence rate of KCOT in 2012 identified six different treatment methods: recurrence rates of 0% were found with resection and enucleation with peripheral ostectomy and subsequent application of CS, as compared to a rate of 50% for enucleation and CS application only. This clearly shows that OKCs should be treated as tumors, even though in many cases they may resemble a unilocular cyst. The fact that OKC recurrence is caused by fragmented enucleation is universally recognized as the probable cause of such high recurrence rates observed in cases of marsupialization. The second most probable source of recurrence is daughter cysts adhered to the walls of the pericystic cavity and mucosa overlying the lesion, particularly when the mucous membrane is overlooked during the surgical procedure. This could explain the high recurrence rates in some studies [19,20]. In a study by Levorova et al. [20], who noted a 45.4% recurrence rate after surgical treatment followed by CS application, the authors admitted that they did not consider the surrounding tissues, which might have been the probable cause the high recurrence rate. 

A retrospective cohort study by Caminiti et al. [21] compared the results of patients treated with 5-fluorouracil (5-FU) cream to cases in which MC was applied: no recurrences were observed in the 5-FU group, as compared to nine recurrences in the MC arm, which constituted a 25% recurrence rate. We analyzed this intervention more closely: the probable reason for such a high recurrence rate in the MC group, which served as a control, may relate to the treatment technique. In the case of 5-FU application, a gauze coated with 5% 5-FU cream was packed into the cavity and removed after 24 h, which allowed for contact with the surrounding soft tissues and thus the possible elimination of offshoots located in the mucosa. In the case of the MC arm, the surrounding tissues were protected with Vaseline post-solution application. We do not agree with the authors that CS or MC application significantly increases the time of surgery, particularly as the 5-FU-coated gauze must be removed 24 h post-surgery and an immediate post-operative orthopantomography X-ray is necessary to ensure proper placement in the cavity, which might prove difficult in cases of uncooperative patients, such as children or mentally disabled patients. 

Our results were consistent with the opinion of Hellstein et al. [22], who explored the penetration values of CS and nonchloroform MC in various types of tissue, to determine the necessity and effect of using chloroform in the cauterizing solution. Their study showed almost identical penetration values for all researched tissue types and concluded that chloroform is a redundant component of CS. 

One of the limitations of this study, besides those universally attributed to retrospective research, is that it addressed the results of a single study center. However, we used a great degree of caution when designing the analysis to avoid bias. An additional constraint would be the relatively shorter follow-up period for the MC arm, since OKCs are known to recur even after as long as 25 years post-operatively [23]. We cannot completely rule out the possibility of further recurrence without using extended postoperative observation. Another limitation might be the lack of a control group in which no chemical cautery was used. This is because adjunct chemical cautery is a gold standard in our department for surgical treatment of histopathologically diagnosed OKCs. 

In conclusion, the results of this study suggested that, when used as adjunctive therapy, the application of MC has comparable efficiency to CS in lowering the recurrence rate of OKCs. Prospective clinical trials of CS versus MC may not be relevant, whereas a trial comparing the effectiveness of MC to 5-FU is more advisable. 

## Figures and Tables

**Table 1:** Analyzed clinical parameters in the studied groups.

Variable			Treatment Group				Statistical Test Results
			CS		MC		
			n	%	n	%	
Sex		Female	24	32.9	18	36.7	χ^2^(1) = 0.19; *p* = 0.7
		Male	49	67.1	31	63.3	
Location	Mandible	−	17	23.3	11	22.4	χ^2^(1) = 0.01; *p* > 0.99
		+	56	76.7	38	77.6	
	Maxilla	−	56	76.7	38	77.6	χ^2^(1) = 0.01; *p* > 0.99
		+	17	23.3	11	22.4	
	Anterior	−	53	72.6	36	73.5	χ^2^(1) = 0.01; *p* > 0.99
		+	20	27.4	13	26.5	
	Posterior	−	20	27.4	13	26.5	χ^2^(1) = 0.01; *p* > 0.99
		+	53	52.6	36	73.5	
Clinical presentation at baseline	Asymptomatic	−	26	35.6	18	36.7	χ^2^(1) = 0.02; *p* > 0.99
		+	47	64.4	31	63.3	
	Edema	−	47	64.4	31	63.3	χ^2^(1) = 0.02; *p* > 0.99
		+	26	35.6	18	36.7	
	Pain	−	47	64.4	31	63.3	χ^2^(1) = 0.02; *p* > 0.99
		+	26	35.6	18	36.7	
	Pus discharge	−	55	75.3	37	75.5	χ^2^(1) = 0; *p* > 0.99
		+	18	24.7	12	24.5	
Extraction of adjacent teeth		−	8	11	1	2	χ^2^(1) = 3.41; *p* = 0.08
		+	65	89	48	98	
Bone grafting		−	50	68.5	43	87.8	χ^2^(1) = 6; *p* = 0.02; ø = 0.22
		+	23	31.5	6	12.2	

− Stands for absence and + for presence of the lesion in mandible or maxilla respectively.

**Table 2:** Incidence of recurrences in compared groups.

Month		Arm				Statistical Test Results
		CS		MC		
		n	%	n	%	
3	−	73	100	49	100	-
	+	0	0	0	0	
6	−	73	100	49	100	-
	+	0	0	0	0	
12	−	73	100	48	98	χ^2^(1) = 1.5; *p* = 0.4
	+	0	0	1	2	
24	−	72	98.6	48	98	χ^2^(1) = 0.08; *p* > 0.99
	+	1	1.4	1	2	
36	−	70	95.9	47	95.9	χ^2^(1) = 0; *p* > 0.99
	+	3	4.1	2	4.1	
48	−	72	98.6	48	98	χ^2^(1) = 0.08; *p* > 0.99
	+	1	1.4	1	2	
60	−	73	100	49	100	-
	+	0	0	0	0	
72	−	73	100	49	100	-
	+	0	0	0	0	
84	−	72	98.6	49	100	χ^2^(1) = 0.68; *p* > 0.99
	+	1	1.4	0	0	
96	−	73	100	49	100	-
	+	0	0	0	0	
108	−	73	100	49	100	-
	+	0	0	0	0	
Total number of recurrences		6	8.2%	5	10.2%	-

− Stands for absence and + for presence of the lesion in mandible or maxilla respectively.

**Table 3:** Association of the total number of recurrences in the CS arm with the analyzed variables.

Variable			Total Number of Recurrences in the CS Arm				Statistical Test Results
			0		1		
			n	%	n	%	
Sex		Female	22	32.8	2	33.3	χ^2^(1) = 0; *p* > 0.99
		Male	45	67.2	4	66.7	
Location	Mandible	−	16	23.9	1	16.7	χ^2^(1) = 0.16; *p* > 0.99
		+	51	76.1	5	83.3	
	Maxilla	−	51	76.1	5	83.3	χ^2^(1) = 0.16; *p* > 0.99
		+	16	23.9	1	16.7	
	Anterior	−	49	73.1	4	66.7	χ^2^(1) = 0.12; *p* = 0.66
		+	18	26.9	2	33.3	
	Posterior	−	18	26.9	2	33.3	χ^2^(1) = 0.12; *p* = 0.66
		+	49	73.1	4	66.7	
Clinical presentation at baseline	Asymptomatic	−	22	32.8	4	66.7	χ^2^(1) = 2.75; *p* = 0.18
		+	45	67.2	2	33.3	
	Edema	−	45	67.2	2	33.3	χ^2^(1) = 2.75; *p* = 0.18
		+	22	32.8	4	66.7	
	Pain	−	45	67.2	2	33.3	χ^2^(1) = 2.75; *p* = 0.18
		+	22	32.8	4	66.7	
	Pus discharge	−	52	77.6	3	50	χ^2^(1) = 2.26; *p* = 0.16
		+	15	22.4	3	50	
Extraction of adjacent teeth		−	7	10.4	1	16.7	χ^2^(1) = 0.22; *p* = 0.52
		+	60	89.6	5	83.3	
Bone grafting		−	45	67.2	5	83.3	χ^2^(1) = 0.67; *p* = 0.66
		+	22	32.8	1	16.7	
Treatment	CS	−	−	−	−	−	-
		+	67	100	6	100	
	MC	−	67	100	6	100	-
		+	−	−	−	−	

− Stands for absence and + for presence of the lesion in mandible or maxilla respectively.

**Table 4:** Association of the total number of recurrences in the MC arm with the analyzed variables.

Variable			Total Number of Recurrences in the MC Arm				Statistical Test Results
			0		1		
			n	%	n	%	
Sex		Female	17	38.6	1	20	χ^2^(1) = 0.67; *p* = 0.64
		Male	27	61.4	4	80	
Location	Mandible	−	8	18.2	3	60	χ^2^(1) = 4.51; *p* = 0.03; ø = 0.3
		+	36	81.8	2	40	
	Maxilla	−	36	81.8	2	40	χ^2^(1) = 4.51; *p* = 0.03; ø = 0.3
		+	8	18.2	3	60	
	Anterior	−	33	75	3	60	χ^2^(1) = 0.52; *p* = 0.6
		+	11	25	2	40	
	Posterior	−	11	25	2	40	χ^2^(1) = 0.52; *p* = 0.6
		+	33	75	3	60	
Clinical presentation at baseline	Asymptomatic	−	15	34.1	3	60	χ^2^(1) = 1.3; *p* = 0.34
		+	29	65.9	2	40	
	Edema	−	29	65.9	2	40	χ^2^(1) = 1.3; *p* = 0.34
		+	15	31.4	3	60	
	Pain	−	29	65.9	2	40	χ^2^(1) = 1.3; *p* = 0.34
		+	15	31.4	3	60	
	Pus discharge	−	35	79.5	2	40	χ^2^(1) = 3.8; *p* = 0.09
		+	9	20.5	3	60	
Extraction of adjacent teeth		−	1	2.3	0	0	χ^2^(1) = 0.12; *p* = 1
		+	43	97.7	5	100	
Bone grafting		−	38	86.4	5	100	χ^2^(1) = 0.78; *p* = 1
		+	6	13.6	0	0	
Treatment	CS	−	24	54.5	3	60	χ^2^(1) = 0.05; *p* = 1
		+	20	45.5	2	40	
	MC	−	20	45.5	2	40	χ^2^(1) = 0.05; *p* = 1
		+	24	54.5	3	60	

− Stands for absence and + for presence of the lesion in mandible or maxilla respectively.

## Data Availability

The data presented in this study are available upon request from the corresponding author. The data are not publicly available since certain data are subject to further research.
